# Navigating fertility care in the telehealth era: association of consultation mode with patient engagement and pregnancy outcomes

**DOI:** 10.1007/s10815-025-03620-4

**Published:** 2025-08-19

**Authors:** Stephanie Willson, Leah Roberts, Thomas Molinaro, Kassie Bollig

**Affiliations:** 1 IVIRMA Global Research Alliance, IVIRMA New Jersey - Reproductive Medicine Associates of New Jersey, 140 Allen Road, Basking Ridge, NJ 07902 USA; 2 Sidney Kimmel College of Medicine, Thomas Jefferson University, Philadelphia, PA USA; 3 Fertility Specialists Network, Boca Raton, FL USA; 4 Robert Wood Johnson Medical School, New Brunswick, NJ USA

**Keywords:** Patient engagement, Telehealth, Assisted reproductive technology, Pregnancy, Infertility

## Abstract

**Purpose:**

To examine levels of patient engagement in fertility care by evaluating the association between initial visit type (in-person versus telehealth) and discharge with an ongoing pregnancy.

**Methods:**

Retrospective study of all new patient visits (*n* = 5527) at an academic fertility clinic from April 2020 to March 2021. The primary outcome was ongoing pregnancy with discharge to obstetrical care, stratified by treatment type: non-in vitro fertilization (IVF) treatment versus IVF. Secondary outcomes included the highest level of patient engagement achieved, defined as the furthest stage in clinical assessment and treatment of infertility, if the primary outcome was not achieved.

**Results:**

A total of 5527 patients were included (telehealth: *n* = 1331, in-person: *n* = 4196). Overall, 44.4% and 47.1% of those who had a telehealth and in-person consultation, respectively, achieved an ongoing pregnancy as a result of any fertility treatment. When stratified by those who utilized non-IVF versus IVF treatment, there was no difference in the probability of discharge with an ongoing pregnancy as a result of treatment after adjusting for age, BMI, time from first visit, AMH, and infertility diagnosis (non-IVF: aOR 1.07, 95% CI 0.73–1.57, *p* = 0.725; IVF: aOR 0.95, 95% CI 0.74–1.22, *p* = 0.688). Those who had an initial in-person visit were 55% more likely to complete diagnostic testing compared to those who had a telehealth visit (telehealth compared to in-person: aOR 0.55, 95% CI 0.45–0.67, *p* < 0.001).

**Conclusions:**

Among those who pursue infertility treatment to conceive, there is no association between initial visit type and ongoing pregnancy as a result of non-IVF or IVF treatment.

## Introduction

Telehealth, or the use of electronic-conferencing platforms to develop communication between a healthcare provider and patient, has been an efficient tool for enhancing patient care. The use of telehealth increased significantly during the COVID-19 pandemic and remains to be utilized by providers nationwide. Indeed, in 2022, approximately 30.1% of adults utilized some aspect of telemedicine within the preceding 12 months, demonstrating that the availability and use of telehealth remains an important means of healthcare access [1]. Studies have demonstrated that different patient factors, such as higher education, female gender, urban area of residence, and private insurance coverage, are associated with higher utilization of telehealth [1].

Telemedicine platforms have been shown to be a cost-effective method to address physician shortages while providing increased access to care in underserved communities where distance to a provider can be a significant barrier [2, 3]. Since the surge in the use of telemedicine platforms in the COVID pandemic, patients have accepted these tools as a part of communication with their healthcare team. A recent survey of patients living in a rural area demonstrated that over half of respondents view telehealth for reproductive health services as a positive aspect of their care [3]. Another study supported the satisfaction with new patient telehealth visits among the reproductive endocrinology patient population. The authors demonstrated that 93% of patients would use a telehealth platform again, 88% reported improved access to care, and 96% endorsed improved travel time, providing evidence that a majority of patients viewed the tool as a way to increase access to care [4].

Patients seeking care for reproductive pathology and fertility evaluation remain in a vulnerable position even prior to their initial visit. Barriers to obtaining care can include limited clinic accessibility, provider availability, and concerns of costs and/or insurance coverage [3]. Once patients do seek reproductive care, the initial assessment of fertility consists of a comprehensive workup, including detailed history gathering, scheduling of laboratory testing, genetic testing, ultrasound evaluation, uterine cavity and fallopian tube assessment, and semen analysis. Patients can oftentimes be overwhelmed during this complex process and be lost to follow-up as a result.

Given that telehealth has been shown to improve access to care, avoid treatment delays, and support clinic operations, the question must be asked if patients who are initially seen for fertility evaluation via telehealth platform have similar subsequent engagement in care and pregnancy outcomes compared to patients seen for an initial in-person appointment. As the nature of fertility care eventually requires patients to be seen in person for the completion of diagnostic testing via physical examination, ultrasound, and other procedures, it is unknown if an initial telehealth visit facilitates a more efficient workup and higher patient engagement in care prior to treatment or, alternatively, serves as a barrier by creating another step in the process. In this study, we aim to examine the levels of patient engagement in fertility care by evaluating the association between initial visit type and discharge with an ongoing pregnancy as a result of fertility treatment.

## Materials and methods

### Study design

This was a retrospective cohort study of patients who had a new patient visit at a single, university-affiliated fertility center between April 2020 and March 2021. All patient data was collected from the electronic medical record (EMR) with exposure status and outcomes manually validated by individual chart review. The primary exposure of this study was initial visit type (in-person versus telehealth). Patients had the autonomy to choose between telehealth with audio and visual capabilities versus an in-person office visit. The study protocol was approved by an institutional review board (Advarra IRB, Pro00027158).

### Study participants

Patients who had previously created embryos or frozen oocytes at an outside clinic, planning oocyte or embryo banking for fertility preservation purposes, or individuals utilizing donor gametes or gestational carriers were excluded. The time frame of April 2020 to March 2021 was chosen due to the change in practice patterns during the COVID-19 epidemic, whereby an increased number of patients were choosing to interact with providers through telehealth. During the study period, in-person consultation continued to be offered with the same availability as prior to the COVID-19 epidemic, allowing an objective comparison between initial patient visit types.

### Data collection

The primary outcome of this study was ongoing pregnancy as a result of fertility treatment with subsequent discharge to obstetrical care at approximately 8 weeks of gestation. Secondary outcomes included the highest level of patient engagement achieved, defined as the furthest stage in clinical assessment and treatment of infertility, if the patient did not achieve the primary outcome. Levels of patient engagement were detailed as follows: achieved unassisted pregnancy during diagnostic testing; did not complete diagnostic testing; completed diagnostic testing but did not pursue treatment; completed diagnostic testing and non-in vitro fertilization (IVF) treatment (ovulation induction with fertility medication (OI) with intrauterine insemination (IUI), OI with timed intercourse (TIC), or IUI alone) but did not achieve a viable pregnancy; completed diagnostic testing and IVF treatment but did not achieve a viable pregnancy. All patients were required to complete diagnostic testing prior to pursuing treatment. If appropriate, patients first utilized non-IVF methods to conceive, followed by IVF if pregnancy was not achieved as part of shared decision-making with their individual provider. Patients who achieved an ongoing pregnancy as a result of non-IVF treatment did not utilize IVF during the study period; patients who achieved an ongoing pregnancy as a result of IVF treatment may or may not have previously utilized non-IVF methods but were ultimately not able to achieve pregnancy with those treatments.

Other important covariates collected for analysis included key patient characteristics such as age at first visit, body mass index (BMI, kg/m^2^), primary infertility diagnosis, day 3 follicle-stimulating hormone (FSH, IU/L), anti-Müllerian hormone (AMH, ng/mL), antral follicle count (AFC), partner status (yes/no), same-sex couple status (yes/no), time of patient follow-up from initial consultation to final pregnancy outcome data extraction (days), and type of fertility treatment (non-IVF versus IVF). Patient age, BMI, FSH, AMH, and AFC were explored as both continuous and categorical variables for descriptive purposes, but continuous categorization was utilized for model building.

### Statistical analysis

This study examined the association between initial fertility visit type and ongoing pregnancy as a result of fertility treatment. Baseline characteristics for those who utilized telehealth versus an in-person visit for initial fertility consultation were compared using Pearson’s chi-square test for categorical variables and the two-sample *t*-test or Wilcoxon rank-sum test for continuous variables that were normally or not normally distributed, respectively. For the primary analysis, multivariable logistic regression was used to identify associations of visit type with discharge with an ongoing pregnancy after adjusting for possible confounders in the entire study population. To account for expected differences in rates of pregnancy by treatment type (non-IVF versus IVF), the fully adjusted model included only those who had utilized fertility treatment to achieve pregnancy to allow stratification for infertility treatment type (non-IVF versus IVF).

Potential confounders were selected a priori based on known biologic plausibility and included maternal age (years), body mass index (BMI, kg/m^2^), primary infertility diagnosis, anti-Müllerian hormone (AMH, ng/mL), partner status (yes/no), same sex couple status (yes/no), and time from initial visit (days). For the primary outcome of ongoing pregnancy as a result of fertility treatment, partner status and same sex status were not included in the model to optimize parsimony. Day three follicle-stimulating hormone (FSH) and antral follicle count (AFC) were also not included due to collinearity with AMH as well as being duplicative measures of ovarian reserve and predicted response to fertility treatment. Because unassisted pregnancy has not been shown to be associated with AMH, this variable was not included in the analysis for this outcome. Additionally, if a patient had an unassisted pregnancy at any time, we did not include these patients in subsequent analyses of stages of patient engagement, as they no longer needed to proceed with care and, therefore, were not able to have these other outcomes. Depending on the model and after excluding patients who no longer could have specific engagement outcomes (i.e., those with unassisted pregnancies), missing covariate values ranged from 7.6 to 12.3%. Given this small range of missingness and our large sample size, we utilized complete case analysis.

All statistical analyses were performed in STATA software version 17.0 (College Station, Texas). A *p*-value of < 0.05 was considered to be statistically significant.

## Results

Between March of 2020 and April of 2021, 5527 patients underwent new patient consultations. Of those, 24.1% (*n* = 1331) chose to engage in a telehealth platform, and 75.9% (*n* = 4196) elected to come into the office for an in-person visit. Baseline patient characteristics are described in Table 1. Patients who chose telehealth had a lower BMI (25.4 versus 26.0 kg/m^2^, *p*-value = 0.006), higher day 3 FSH (7.3 versus 7.1 IU/L, *p*-value = 0.018), lower AMH (2.4 versus 2.8 ng/mL, *p*-value < 0.001), and a lower AFC (15 versus 16, *p*-value = 0.003). Those who chose an initial in-person consultation were less likely to have diagnoses of diminished ovarian reserve (22.4% versus 24.6%) or “other” (14.2% versus 16.5%) but had higher rates of polycystic ovarian syndrome diagnosis (20.2% versus 13.4%) (*p*-value < 0.001). Additionally, individuals who had an in-person first visit had a shorter time of follow-up compared to those who had a telehealth visit (782 versus 926 days, *p*-value < 0.001). There was no difference in whether patients had a partner or were part of a same-sex couple.

**Table 1:** Baseline characteristics

	Total *n* = 5527	In-person *n* = 4196	Telehealth *n* = 1331	*p*-value
Age (years)	35.1 (5.0)	35.0 (5.0)	35.3 (5.0)	0.085
SART age categories (years)				0.38
< 35	2753 (49.8%)	2097 (50.0%)	656 (49.3%)	
35–37	1201 (21.7%)	931 (22.2%)	270 (20.3%)	
38–40	871 (15.8%)	648 (15.4%)	223 (16.8%)	
41–42	2753 (49.8%)	2097 (50.0%)	656 (49.3%)	
> 42	1201 (21.7%)	931 (22.2%)	270 (20.3%)	
Body mass index (kg/m^2^)	25.8 (22.5–31.0)	26.0 (22.6–31.3)	25.4 (22.3–30.0)	**0.006**
Body mass index (categorical, kg/m^2^)				**0.017**
Optimal (18.5– < 25)	2221 (40.2%)	1723 (41.1%)	498 (37.4%)	
Underweight (< 18.5)	104 (1.9%)	87 (2.1%)	17 (1.3%)	
Overweight (25– < 30)	1437 (26.0%)	1131 (27.0%)	306 (23.0%)	
Obese class I (30– < 35)	795 (14.4%)	642 (15.3%)	153 (11.5%)	
Obese class II (35– < 40)	406 (7.3%)	334 (8.0%)	72 (5.4%)	
Obese class III (40– < 42)	100 (1.8%)	82 (2.0%)	18 (1.4%)	
Obese class III (≥ 42)	212 (3.8%)	183 (4.4%)	29 (2.2%)	
Missing	252 (4.6%)	14 (0.3%)	238 (17.9%)	
Primary infertility diagnosis				** < 0.001**
DOR	1265 (22.9%)	938 (22.4%)	327 (24.6%)	
Endocrine	53 (1.0%)	43 (1.0%)	10 (0.8%)	
Endometriosis	123 (2.2%)	94 (2.2%)	29 (2.2%)	
Genetic	118 (2.1%)	77 (1.8%)	41 (3.1%)	
Male factor	1159 (21.0%)	895 (21.3%)	264 (19.8%)	
Other	817 (14.8%)	597 (14.2%)	220 (16.5%)	
PCOS	1025 (18.5%)	846 (20.2%)	179 (13.4%)	
RPL	347 (6.3%)	246 (5.9%)	101 (7.6%)	
Uterine	136 (2.5%)	111 (2.6%)	25 (1.9%)	
Tubal factor	270 (4.9%)	201 (4.8%)	69 (5.2%)	
Missing	214 (3.9%)	148 (3.5%)	66 (5.0%)	
Day 3 follicle-stimulating hormone (IU/L)	7.1 (5.8–8.8)	7.1 (5.8–8.7)	7.3 (5.9–9.0)	**0.018**
Day 3 follicle-stimulating hormone (categorical, IU/L)				0.066
Normal (≤ 10)	3620 (65.5%)	2824 (67.3%)	796 (59.8%)	
Abnormal (> 10)	708 (12.8%)	530 (12.6%)	178 (13.4%)	
Missing	1199 (21.7%)	842 (20.1%)	357 (26.8%)	
Anti-Müllerian hormone (ng/mL)	2.7 (1.2–5.1)	2.8 (1.2–5.3)	2.4 (1.1–4.34)	** < 0.001**
Anti-Müllerian hormone (categorical, ng/mL)				** < 0.001**
Normal (1.0–3.0)	1694 (30.6%)	1278 (30.5%)	416 (31.3%)	
Very low (< 0.5)	518 (9.4%)	388 (9.2%)	130 (9.8%)	
Low (0.5– < 1.0)	543 (9.8%)	394 (9.4%)	149 (11.2%)	
High (> 3.0)	2324 (42.0%)	1843 (43.9%)	481 (36.1%)	
Missing	448 (8.1%)	293 (7.0%)	155 (11.6%)	
Antral follicle count	15.0 (9.0–24.0)	16.0 (10.0–24.0)	15.0 (9.0–22.0)	**0.003**
Antral follicle count (categorical)				**0.016**
Normal (10–20)	2013 (36.4%)	1575 (37.5%)	438 (32.9%)	
Low (≤ 10)	1540 (27.9%)	1196 (28.5%)	344 (25.8%)	
High (> 20)	1649 (29.8%)	1343 (32.0%)	306 (23.0%)	
Missing	325 (5.9%)	82 (2.0%)	243 (18.3%)	
Partner status				0.58
No	170 (3.1%)	126 (3.0%)	44 (3.3%)	
Yes	5357 (96.9%)	4070 (97.0%)	1287 (96.7%)	
Same-sex couple status				0.51
No	5214 (94.3%)	3958 (94.3%)	1256 (94.4%)	
Yes	143 (2.6%)	112 (2.7%)	31 (2.3%)	
Missing	170 (3.1%)	126 (3.0%)	44 (3.3%)	
Time of patient follow-up (days)	802.0 (712.0–891.0)	782.0 (703.0–850.0)	926.0 (773.0–962.0)	** < 0.001**

Data are presented as mean (SD) or median (IQR) for continuous measures and *n* (%) for categorical measures

Bold indicates statistically significant *p*-values < 0.05

**Fig. 1:**
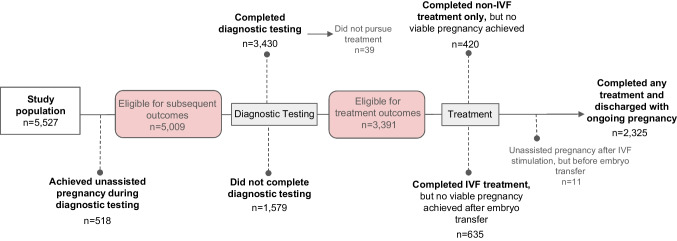
Patient engagement timeline. IVF, in vitro fertilization

**Fig. 2:**
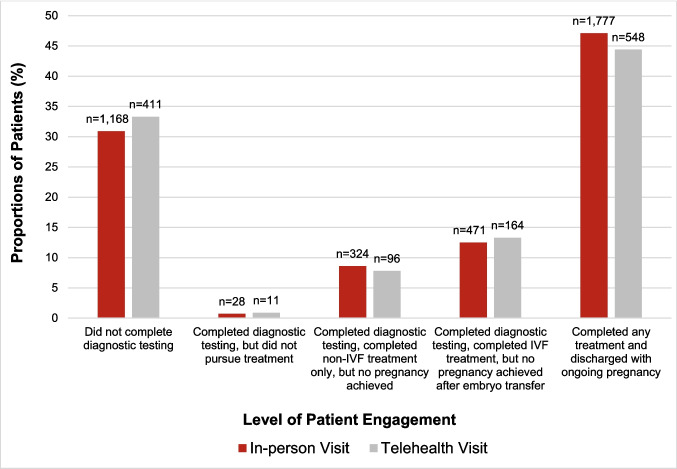
Proportions of patients for each level of engagement in those with initial telehealth (*n* = 1234) versus in-person (*n* = 3775) appointments. IVF, in vitro fertilization

Figure 1 describes the total number of patients completing each stage of patient engagement. Seven percent (*n* = 97) of individuals who had an initial telehealth visit and 10.0% (*n* = 421) of those who had an initial in-person visit achieved an unassisted pregnancy during diagnostic testing (aOR 0.78, 95% CI 0.59–1.03, *p* = 0.081) and were excluded from subsequent analyses. Of the remaining patients (*n* = 5009), similar proportions of patients who had an initial in-person visit versus telehealth visit were seen at each level of patient engagement (all *p*-values > 0.05; Fig. 2). Overall, 44.4% (*n* = 548) of those who had a telehealth consultation and 47.1% (*n* = 1777) of those who had an in-person consultation completed diagnostic testing and achieved an ongoing pregnancy as a result of fertility treatment. Among all patients, individuals who had an initial visit via telehealth were 18% more likely to complete diagnostic testing, any fertility treatment, and achieve an ongoing pregnancy compared to those who had an in-person consultation when adjusting for maternal age, BMI, time from first visit, AMH, and infertility diagnosis (aOR 1.18, 95% CI 1.00–1.40, *p*-value = 0.047). However, when stratifying by treatment type in fully adjusted models, this difference was attenuated. In individuals who utilized IVF, there was no difference in the probability of achieving an ongoing pregnancy between those who had an initial in-person consult versus those who had an initial telehealth visit after adjusting for maternal age, BMI, time from first visit, AMH, and infertility diagnosis (aOR 0.95, 95% CI 0.74–1.22, *p*-value 0.688). For those who utilized non-IVF methods only to conceive, there was also no difference in the probability of achieving an ongoing pregnancy between those who had an initial in-person consult versus those who had an initial telehealth visit after adjusting for age, BMI, time from first visit, AMH, and infertility diagnosis (aOR 1.07, 95% CI 0.73–1.57, *p* = 0.725).

Table 2 shows adjusted analyses of all patient engagement outcomes. Compared to those who had an initial in-person visit, those who had telehealth were 45% less likely to not complete diagnostic testing (aOR 0.55, 95% CI 0.45–0.67, *p* < 0.001). There were no other statistically significant differences in other levels of patient engagement in fully adjusted models.

**Table 2:** Adjusted analyses of all patient engagement outcomes in the telehealth visit group compared to the in-person visit group

	Adjusted odds ratio	95% CI	*p*-value
Primary outcome			
Complete diagnostic testing, non-IVF fertility treatment, and achieved an ongoing pregnancy^a^	1.07	0.73–1.57	0.725
Complete diagnostic testing**,** IVF fertility treatment, and achieved an ongoing pregnancy^a^	0.95	0.74–1.22	0.688
Secondary outcomes			
Achieved unassisted pregnancy during diagnostic testing^b^	0.78	0.59–1.03	0.081
Did not complete diagnostic testing^c^	0.55	0.45–0.67	< 0.001
Completed diagnostic testing, but did not pursue treatment^c^	1.96	0.90–4.30	0.090
Completed diagnostic testing, completed non-IVF treatment only, but no pregnancy achieved^a^	1.01	0.77–1.33	0.927
Completed diagnostic testing, completed IVF treatment, but no pregnancy achieved after embryo transfer^a^	1.20	0.96–1.50	0.103

*IVF* in vitro fertilization

^a^Adjusted for maternal age, body mass index, time since first consult, infertility diagnosis, anti-Müllerian hormone level

^b^Adjusted for maternal age, body mass index, time since first consult, infertility diagnosis, partner status, same-sex status

^c^Adjusted for maternal age, body mass index, time since first consult, infertility diagnosis

## Discussion

In this study exploring the impact of initial patient visit type, patients who completed telehealth consultations were more likely to complete diagnostic testing and achieve an ongoing pregnancy when compared to an in-person visit when evaluating all treatment cycles. However, after accounting for important differences in pregnancy rates by stratifying by treatment type (IVF versus non-IVF), the difference in ongoing pregnancy between each group was not statistically significant. When evaluating other stages of patient engagement, we demonstrated that those who had an initial telehealth consultation were more likely to complete diagnostic testing compared to those with an in-person consultation; no differences were seen examining other stages of patient engagement. These findings suggest that the incorporation of telemedicine into routine clinical practice does not negatively impact the probability of a patient achieving an ongoing pregnancy, whether achieved via non-IVF techniques or with IVF. Furthermore, telehealth may enhance certain aspects of patient engagement, specifically initiation of initial fertility testing and workup through a diagnostic cycle.

Our study’s results align with prior data demonstrating that patients seeking reproductive care have similar satisfaction rates using telehealth compared to in-person visits [5]. In addition to patient satisfaction, convenience and access to fertility care remain a major factor in patient engagement given the complex, multi-step nature of treatment options [6]. A study by Alexander et al. found that patients who live farther from clinics and have longer durations of infertility may be more likely to use telehealth to establish care in the infertility setting [7]. In agreement with the findings in our study, this retrospective cohort study of 140 patients (*n* = 70 for telemedicine and in-person visits) also found that there was no difference between groups in the percent of patients that received treatment or time to pregnancy [7]. Our data suggests that the use of telehealth as an initial communication between a patient and their provider may overcome some of the challenges patients may experience when establishing care and promote subsequent engagement in diagnostic evaluation and treatment. Previous studies assessing rapid telemedicine implementation have shed light on access and quality gaps within the medical field [8]. This study adds to the literature to target interventions and quality improvement to keep patients engaged in their care and stay active in treatment and ongoing management. Furthermore, this data supports continued insurance reimbursement for telehealth visits to ensure equitable access to care, allowing patients to benefit from remote consultations without additional financial barriers.

In 2024, the American Society for Reproductive Medicine (ASRM) published an ethics committee document highlighting considerations for telemedicine delivery of fertility care [9]. In this document, ASRM emphasizes the potential for telemedicine to enhance patient access to medical care while at the same time noting ethical concerns. A potential negative effect noted in this committee opinion includes the lack of physical examination and delivering lower quality care. On the contrary, our study demonstrates that completion of an initial visit via a telehealth platform does not sacrifice ongoing pregnancy rates or patient engagement in care during the diagnostic testing phase. Furthermore, our study supports that telehealth can be used as a tool by clinics to reduce the likelihood of incomplete diagnostic testing, which aligns with the field’s goals to improve access to care and reduce burdens and barriers to initiating fertility treatment. By initially reviewing the diagnostic testing plan in its entirety, this may facilitate the patient’s ability to complete the diagnostic phase in fewer visits overall (i.e., knowledge of fasting status or cycle timing for lab work or imaging).

Our study poses several strengths. During the study time period early in the COVID-19 pandemic, patients were highly motivated to access medical care remotely but still maintained the ability to receive in-person care and evaluation if desired, allowing for equal accessibility to either type of initial consultation. This permitted patients to choose their preferred method of initial contact while providing an exchangeable comparator cohort of patients who participated in subsequent evaluation and treatment steps in the same external and political environment during the same time period. Even in this setting of uncertainty during the early stages of the pandemic, these data demonstrate that patients who initially sought out care via telehealth were still motivated to complete their in-person diagnostic workup via in-person physical evaluation, laboratory testing, and further imaging studies. Additionally, the nature of the practice where the study was performed allowed for a large sample size to be studied over a short period of time while minimizing changes in practice patterns or policies that could subsequently affect levels of patient engagement from initiation of care to discharge. Because levels of patient engagement may significantly vary according to the treatment plan, it was also important to analyze results by patient treatment type and provide conclusions for patients who may require fewer visits and procedures (non-IVF treatment cycles) and for those who utilize in-person assessment and evaluation at more frequent intervals (IVF treatment cycles). Previous survey-based investigations examining patients’ views on the use of telehealth in the reproductive care setting have been limited by low response rates (approximately 30%) and an overall low number of participants (*n* = 80–100) [4, 10]. This study extends our knowledge from solely assessing patient acceptability of telemedicine to examining if this mode of communication affects subsequent patient engagement and pregnancy outcomes—the overall goal of our field. Finally, this study compared in-person visits to the actual telehealth platform that has been increasingly integrated into the clinical setting nationwide after the COVID-19 pandemic, allowing for generalization of study findings of tools available for patient engagement to current practice.

There are also limitations in this study to acknowledge. This study was conducted at a single center in a region with state-mandated fertility coverage. As a result, patients in this study may have “better” insurance coverage for diagnostic testing and treatment and are more motivated to complete pending workups compared to those without any fertility coverage or who reside in regions with lesser forms of coverage. Also of note, similar to medical documentation practices of other large-scale clinics, certain data in our electronic medical records rely on accurate and updated input from individual providers. However, for patients to successfully advance from diagnostic to treatment phases of care, standardized checklists were utilized to ensure a complete diagnostic workup prior to treatment initiation. Additionally, our primary outcome of being discharged with an ongoing pregnancy was confirmed by laboratory and ultrasound evaluation. Duration of infertility and parity were not included in our models due to these data not being reliably collected in our EMR. However, we anticipate these characteristics were equally distributed in both exposure groups as neither is likely to have an association with a patient choosing between telehealth or in-person for an initial visit. Lastly, the limitations that accompany a retrospective cohort design must be acknowledged when interpreting these findings. However, the retrospective nature of this study allowed interpretation of results during a unique time of increased utilization and evolution of telemedicine due to the COVID-19 pandemic.

## Conclusion

The use of a telehealth platform as an initial form of patient contact in fertility care compared to in-person consultation did not significantly impact rates of ongoing pregnancy after stratification by fertility treatment type (IVF versus non-IVF) and may enhance diagnostic testing and evaluation completion rates. Our findings suggest that the adoption of a telehealth system by fertility clinics can be used as a tool to increase patient engagement in those initiating infertility evaluation while not compromising outcomes. Together, our data provide real-world evidence for continued use and adaptation of telemedicine to increase patient access to care and advance patient-centered transformation of clinical fertility practice. Future directions include the need for assessment of cost–benefit analyses and extension of telehealth utilization for other points of patient contact, including education and treatment instruction, that may further improve patient understanding, comfort, and clinical efficiency.

## Data Availability

Data regarding any of the subjects in the study has not been previously published. Data will be made available to the editors of the journal for review or query upon request pre and/or post publication. The appropriate (STROBE) checklist for this study design was followed.
